# Dose finding of OpunDia^TM^ (O. ficus-indica extract) for its effect on oral glucose tolerance and plasma insulin

**DOI:** 10.1186/1550-2783-9-S1-P25

**Published:** 2012-11-19

**Authors:** Ivo Pischel, Karen Van Proeyen, Peter Hespel

**Affiliations:** 1 PhytoLab GmbH & Co. KG, Vestenbergsgreuth, Germany; 2 Research Center for Exercise and Health, Department of Biomedical Kinesiology, Faculty of Kinesiology and Rehabilitation Sciences, K.U. Leuven, Belgium

## Background

High-intensity exercise typically leads to a depletion of body carbohydrate stores, primarily muscle glycogen. Therefore, typical ‘sports recovery drinks’ include a high carbohydrate dose together with proteins so as to stimulate muscle glucose uptake and glycogen resynthesis via increased plasma insulin level. In fact, any intervention that elevate plasma insulin following exercise could facilitate repletion of muscle glycogen stores, and serve as a useful ‘recovery agent’. Extracts of the prickly pear cactus (Opuntia ficus-indica; OFI) can stimulate insulin secretion [1], but the most effective dose was not yet elucidated.

## Methods

A double-blind randomized cross-over study was performed. Five subjects participated in four experimental sessions after a 10-12 hr overnight fast with a 1-week interval in between. They received either 500, 1000 or 1500 mg of encapsulated OFI-extract (OpunDia^TM^, an aqueous extract of OFI; Finzelberg GmbH & Co. KG, Germany), or placebo capsules (LUVOS Heilerde) with identical appearance. Thirty min after ingestion of the capsules, a 2-hr oral glucose tolerance test (OGTT: 75g of glucose in 300ml water; blood samples (5ml) at 0, 30, 60, 90, and 120 min) was started. Plasma samples were assayed for glucose and insulin concentration. Student's paired T-tests were used to evaluate treatment effects. A probability level (p) < 0.05 was considered statistically significant.

## Results

Compared with placebo, the area under the serum insulin curve in the OGTT was significantly lower (p<0.05) at 1000 and 1500 mg OFI, but not in 500mg OFI. Administration of OFI in a dose of 1000 mg increased serum insulin concentration throughout the OGTT about two-fold compared with placebo, but no further increase occurred at an even higher dose (1500mg).

Compared with placebo, the area under the blood glucose curve (AUC) was not significantly decreased after oral administration of either 500, 1000 or 1500 mg of encapsulated OFI-extract. The lowest value was found at 1000 mg of OFI with a drop (n.s.) of about -14% compared to placebo.

**Figure 1:**
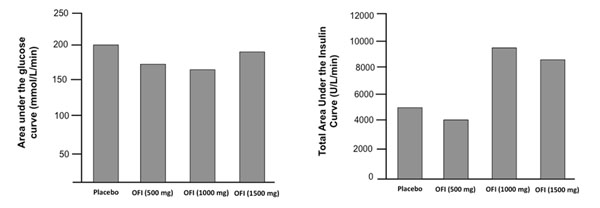


## Conclusions

It was previously shown that the aqueous extract of OFI stimulates insulin secretion before and after endurance exercise bouts (although not significant) and lowered the blood glucose level in young healthy sportsmen. The current study identifies the most effective dose of OFI to stimulate post exercise insulin secretion to be 1000mg of aqueous extract of prickly pear (OpunDia^TM^). It may be a promising and safe ingredient for the development of dietary and sports supplements with insulin secreting activity. Thus, OpunDia^TM^ might act as a “recovery agent” to stimulate post exercise muscle glycogen and protein resynthesis. Additional studies are requested to test the hypothesis that ingestion of OFI-extract together with carbohydrates can stimulate post-exercise muscle glycogen resynthesis, indeed.
